# Deep neck infection and descending mediastinitis as lethal complications of dentoalveolar infection: two rare case reports

**DOI:** 10.1186/s13256-018-1724-x

**Published:** 2018-07-07

**Authors:** Bamidele Johnson Alegbeleye

**Affiliations:** Department of Surgery, Banso Baptist Hospital, P.O Box 9, Nso-Kumbo, Northwestern Region Cameroon

**Keywords:** Case report, Dentoalveolar abscess, Descending mediastinitis

## Abstract

**Background:**

We report two cases of innocuous dentoalveolar infections which rapidly progressed to deep neck abscesses complicated by descending mediastinitis in a resource-constrained rural mission hospital in the Cameroon.

**Case presentation:**

The clinical presentations of a 35-year-old man and a 32-year-old woman both of Fulani origin in the Northern region of Cameroon were similar with submandibular fluctuant and tender swelling and differential warmth to palpation. The patients had tachycardia, high grade pyrexia, and normal blood pressure. Further physical and neurological examinations were unremarkable. An ultrasound scan of the neck swellings showed submandibular turbid collections. Plain chest radiographs confirmed empyema thoraces. Our patients had serial drainage of the neck abscesses as well as closed thoracostomy tube drainage which were connected to pleurovac and suctioning machines, with significant amount of pus drainage. Both patients were admitted to our intensive care unit for close monitoring. The first patient continued to make satisfactory clinical progress and was discharged by the fourth week of admission. The patient who had human immunodeficiency viral infection died on the fifth postoperative day.

**Conclusions:**

The possibility of lethal complications and the associated morbidity and mortality portray this clinical entity as an important public health concern. Clinicians taking care of patients with dentoalveolar and oropharyngeal infections need to be sensitized to these potentially fatal complications. *Alternatively, strategies to improve oral health and reduce the incidence of dental caries, the main cause of dental abscess, would maximize use of resources; especially in resources-constrained centers like ours in Banso Baptist Hospital.*

## Background

Acute dental abscess usually occurs secondary to dental caries, trauma, or failed root treatment [1]. Dentoalveolar infections are one of the most common diseases in the oral and maxillofacial region [2, 3]. Complications are associated with a mortality rate of 10–40% [4]. With the advent of modern antibiotics, mortality rates have significantly reduced [5, 6]. Multiple severe complications of dentoalveolar infection have been reported, such as airway obstruction, Ludwig angina, descending mediastinitis, necrotizing fasciitis, cavernous sinus thrombosis (CST), sepsis, thoracic empyema, cerebral abscess, and osteomyelitis [7–9]. Most oropharyngeal infections are self-limiting and contained. However, they can spread through the fascia and deep neck spaces while progressing inferiorly into the mediastinum, especially in the diabetic, immunocompromised, or debilitated patient [10–12]. We report two interesting cases of seemingly innocuous dentoalveolar infections which rapidly progressed to deep neck abscesses complicated by descending mediastinitis, which are life-threatening infections in resource-limited settings, such as Banso Baptist Hospital, Cameroon.

## Case presentation

### Case 1

**Fig. 1:**
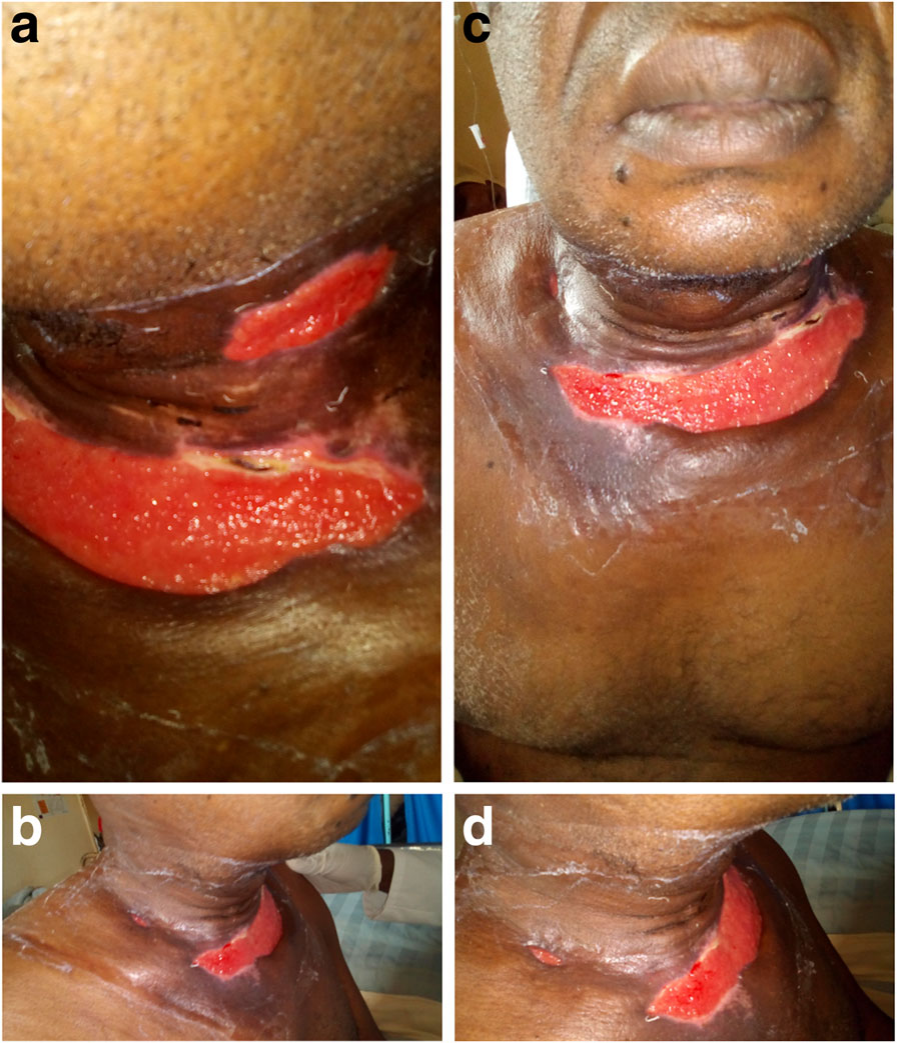
**a**–**d** Post incision and drainage (I & D)

**Fig. 2:**
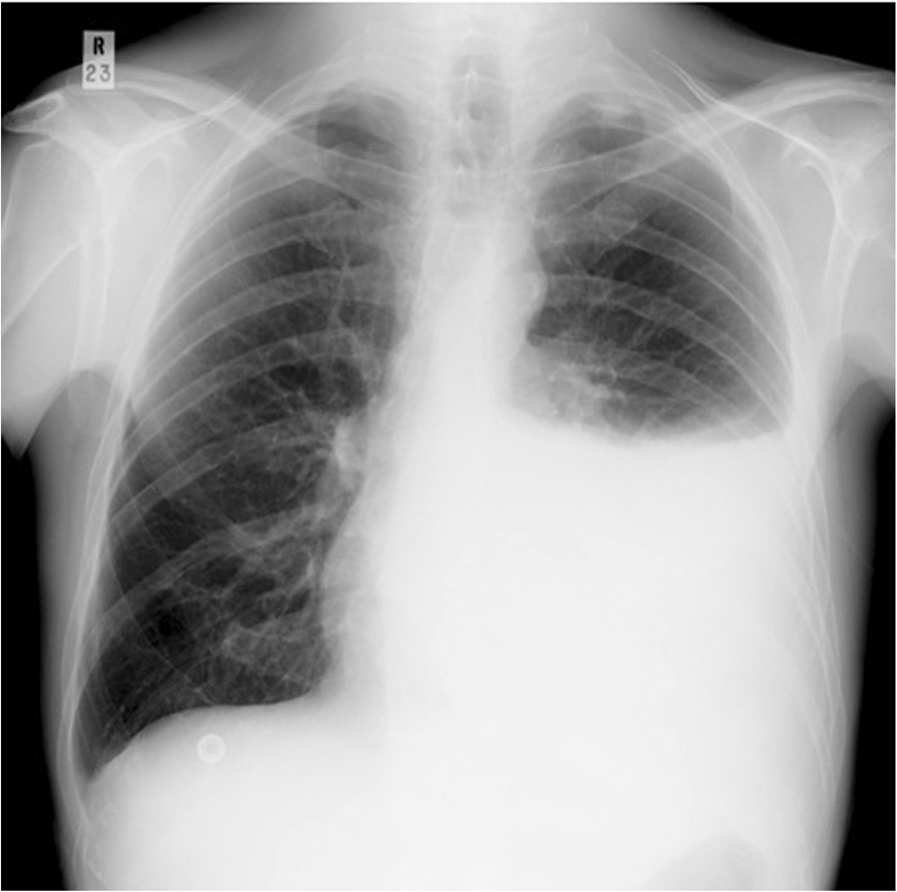
Left Empyema Thoracic

A 35-year-old man of Fulani origin in the Northern region of Cameroon, previously healthy, presented to our surgical ward with severe left submandibular non-fluctuant swelling, which was erythematous and warm to palpation. Furthermore, mild trismus and sublingual edema were noted. Otherwise, physical and neurological examinations were unremarkable. He had a sinus tachycardia of 110 beats/minute, fever 39 °C, and blood pressure 140/85 mmHg. A chest radiograph on admission was normal. He had a 1-month history of a left mandibular second and third molar teeth untreated infection (periodontitis). He was urgently transferred to our operating room (OR) where he underwent surgical drainage of the left submandibular abscess; he was then admitted to our intensive care unit (ICU) while sedated, mechanically ventilated, hemodynamically stable, and febrile (38.7 °C). Laboratory tests were noted for extensive leukocytosis (30,000 cells/ul). Immediately on ICU admission, broad-spectrum parenteral antibiotic therapy (intravenously administered ceftriaxone 1 gm 12 hourly and intravenously administered metronidazole 500 mg 8 hourly) was initiated. The following day, extensive bilateral submandibular abscess collection (left >> right) and multiple cervical lymphadenopathy were shown on an urgently performed superficial ultrasonography scan and he was transferred to our OR again for exploration of deep neck abscess. He was co-managed by the dental surgery team. On the second day of admission, he had extraction of the left mandibular second and third molar teeth by the dental surgeon. A large amount of purulent fluid was drained; 3 days later cultures were positive for mixed aerobic (Gram-positive cocci, commonly streptococci) and anaerobic (*Bacteroides* species essentially *Peptostreptococcus* species) bacteria. He remained febrile (39.3 °C) with a white blood cell (WBC) count of 24.5, although hemodynamically stable. An additional chest radiograph and ultrasound scan of the soft tissue neck and chest 2 days later showed minimal collection in the anterior superior mediastina space and significant abscess in both pleural spaces (empyema thoracis), which was far greater in his left hemithorax. An urgent left closed thoracostomy tube drainage (CTTD) was performed and connected to pleurovac and suction. A large amount (approximately 800 cc) of purulent fluid, pH 7.18, was drained from his left pleural space; he was transferred back to the ICU. Bacterial culture samples of serous fluid were positive again for mixed microbial flora as above. He continued intravenously administered antibiotics therapy with ceftriaxone, ciprofloxacin, and metronidazole only due to bacterial susceptibility of isolated growth of polymicrobial flora. Figure 1 is a post-incision and drainage photograph of this patient showing multiple neck wounds and sinuses. Figure 2 is a plain chest radiograph showing the left thoracic empyema. During the next 4 days he went to OR two additional times for surgical debridement, drainage, and washout of neck wounds. He continued the neck wound washout and antibiotic therapy over the next 3 weeks. He was discharged from the unit 4 weeks after admission.

### Case 2

A 32-year-old woman of Fulani origin in the Northern region of Cameroon presented to our surgical ward with a 5-day history of a painful right submandibular swelling with involvement of the right side of her neck and upper anterior chest wall. There was associated right upper quadrant abdominal pain. These symptoms were preceded by 1-week history of right second and third mandibular teeth infection which was left untreated. She was newly diagnosed with retroviral infection but yet to commence highly active antiretroviral treatment (HAART) before admission. She had right submandibular fluctuant and tender swelling, which was warm to palpation. Further physical and neurological examinations were unremarkable. She had tachypnea 32 breaths/minute, tachycardia 140 beats/minute, fever 38.5 °C, and blood pressure 120/70 mmHg. An ultrasound scan of the submandibular and neck swelling showed right submandibular turbid collection with inflamed muscles. A chest radiograph revealed blunting of the right costophrenic angle. Thoracocentesis revealed a pleural fluid analysis of marked leukocytosis 57,000 cells/ul, predominantly Gram-positive cocci.

**Fig. 3:**
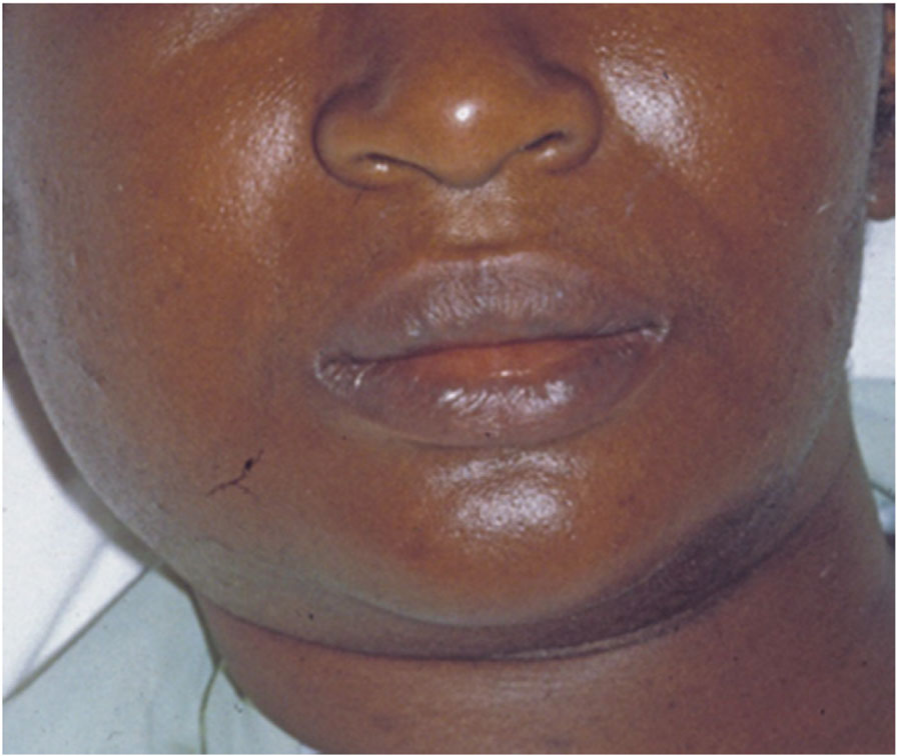
Pre- incision and drainage photograph

**Fig. 4:**
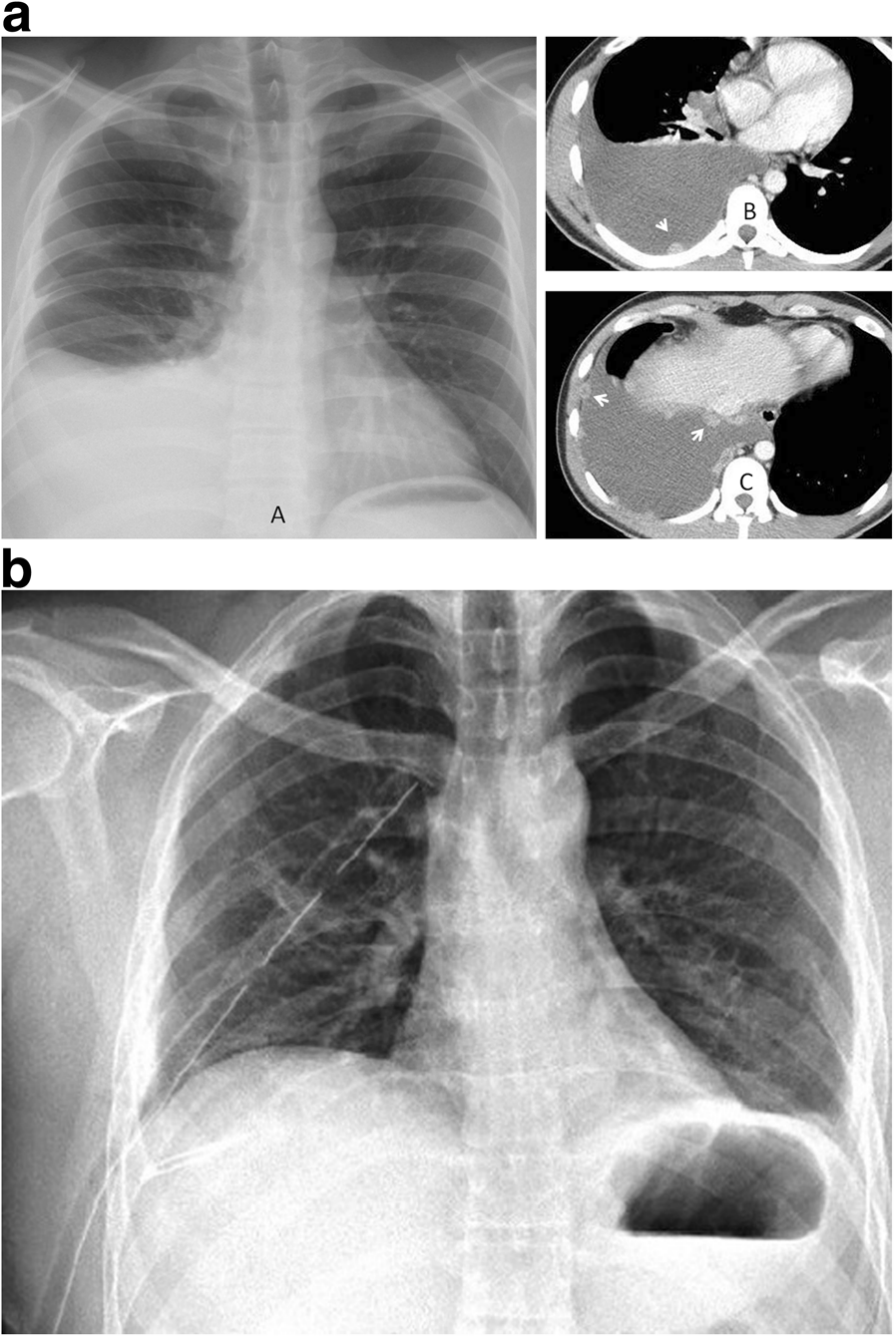
**a** Radiograph & computed tomography (CT). **b** Post chest tube radiograph

She was urgently transferred to our OR where she underwent surgical drainage of the right submandibular abscess and right CTTD connected to pleurovac and suction; she was then admitted to our ICU while sedated, ventilated on oxygen by facemask, hemodynamically stable, and febrile (38.7 °C). A laboratory test was notable for extensive leukocytosis (17,000 cells/ul). Immediately on ICU admission broad-spectrum parenteral antibiotic therapy (intravenously administered ceftriaxone 1 gm 12 hourly and intravenously administered metronidazole 500 mg 8 hourly) was initiated. On the following day, she underwent extensive washout of abscess spaces of the submandibular region and neck. The culture of the submandibular abscess isolated viridans streptococci after 72 hours and the bacteria of the isolated growth were susceptible to Augmentin and doxycycline. Figure 3 is a pre-incision and drainage photograph of this patient showing right submandibular abscess. Figure 4 is a plain chest radiograph and computed tomography (CT) slides showing the left thoracic empyema. Her postoperative period was essentially unremarkable with our patient showing significant relief of symptoms. On the fifth day of admission she developed sudden onset of cardiac arrest from which she could not be resuscitated and was certified dead approximately 1 hour later.

## Discussion

Lethal descending mediastinitis complicating dentoalveolar abscess was a rare presentation to us in Banso Baptist Hospital until now. Our data correlated well with previously published literature. The two patients are of Fulani origin in the Northern region of Cameroon. They are nomadic in nature and feed predominantly on milk and dairy products as staple food with very poor oral hygiene habits. In this case report, both patients had a preexisting history of untreated teeth infections which involved the mandibular molars, due to the proximity of their apices to the submandibular spaces. This may explain a rapid and downward spread of the infectious process which progressed to involve a large part of the neck and anterior mediastinum tissues [13, 14]. In the first index patient the pathogens isolated were of a polymicrobial pattern comprising mixed aerobic (Gram-positive cocci, commonly streptococci) and anaerobic (*Bacteroides* species essentially *Peptostreptococcus* species) bacteria; while the primary and single pathogen isolated in both neck and mediastinum samples of the second index patient was facultative anaerobe viridans group streptococci [13, 14]. The pathogens in both cases are essentially a very common cause of mediastinitis and deep neck infection [13–16]. A complex mix of strict anaerobes and facultative anaerobes account for most infections (59–75%), which can prove challenging to non-specialist microbiology laboratories [17–19]. One literature report described an interesting case of dentoalveolar infection complicated by descending necrotizing mediastinitis [7]. In that case the infectious process was caused by polymicrobial flora (*Streptococcus constellatus* and *Propionibacterium acnes*) [7]. Another author reported two cases of *Propionibacterium* growth (out of 118 patients) in deep space head and neck infections [20]. The poor oral health practices of Cameroonian Fulanis and the delay in presentation are the most significant risk factors for morbidity and mortality in these two patients [21]. The diagnosis of immunosuppressive illness (untreated HIV infection) in the second patient corroborated the fact that an innocuous neck infection in such patients can progress inferiorly with significantly high fatality. An appropriate management of deep neck infection and mediastinitis includes intravenously administered antibacterial therapy and surgical drainage of the cervical and mediastinal collections [16]. In our case immediate, extensive, and recurrent surgical drainage allowed for a successful and early control of the source of infection.

## Conclusions

Finally, clinicians taking care of patients with dentoalveolar and oropharyngeal infections should be sensitized to this potentially fatal complication. Recognition of the classic signs of severe dentoalveolar infections by the general practitioner and expeditious referral to a higher level of care benefits the patient and may be lifesaving. Alternatively, strategies to improve oral health and reduce the incidence of dental caries, the main cause of dental abscess, would maximize use of resources.
